# Arsenic-Based Antineoplastic Drugs and Their Mechanisms of Action

**DOI:** 10.1155/2008/260146

**Published:** 2008-04-09

**Authors:** Stephen John Ralph

**Affiliations:** School of Medical Sciences, Griffith University, Parklands Drive, Southport, Queensland 4215, Australia

## Abstract

Arsenic-based compounds have become accepted agents for cancer therapy providing high rates of remission of some cancers such as acute promyelocytic leukemia (APL). The mechanisms by which arsenic-containing compounds kill cells and reasons for selective killing of only certain types of cancer cells such as APLs have recently been delineated. This knowledge was gained in parallel with increasing understanding and awareness of the importance of intracellular redox systems and regulation of the production of reactive oxygen species (ROS) by controlling mitochondrial function. Many of the targets for the arsenic-containing compounds are mitochondrial proteins involved in regulating the production of ROS. Inhibition of these proteins by disulfide linkage of vicinal thiol groups often leads to increased production of ROS and induction of apoptotic signalling pathways. Sensitivity or resistance to the actions of arsenic-containing compounds on cancer cells and normal cells depends on the levels of transport systems for their uptake or efflux from the cells as well as their redox defence mechanisms. The exact mechanisms of arsenic toxicity as well as its anticancer properties are likely to be related and these aspects of arsenic metabolism are covered in this review. Greater understanding of the mechanisms of action of arsenic will help determine the risks of human exposure to this chemical. Novel organic arsenic-containing compounds and the lessons learned from studying their selective sensitivity in targeting dividing endothelial cells to inhibit angiogenesis raise the future possibility for designing better targeted antineoplastic arsenic-containing compounds with less toxicity to normal cells.

## 1. ARSENIC AND ARSENIC-CONTAINING COMPOUNDS IN THE NATURAL ENVIRONMENT OR AS A RESULT OF HUMAN APPLICATION

Arsenic is a toxic metalloid [1, 2] that exists throughout nature in organic and inorganic forms. It is commonly present in soils on average at several mg/kg, as well as in marine sediments, and is enriched in mineral deposits as oxides and sulfides. The basic element, arsenic, exists in either of three allotropic forms: yellow, black, or grey with the stable, semimetallic form having a silver/steely-grey colour as a brittle, crystalline solid. The semimetallic form oxidises rapidly in air, and at high temperatures produces a white cloud of arsenic trioxide (As_2_O_3_). Arsenic, with its variety of chemical forms and oxidation states, is listed in Table 1. The current IUPAC nomenclature is listed in [3] as well as the more common names of the arsenic-based compounds which are mainly used throughout this review.

When absorbed at toxic levels, arsenic causes severe health problems, including cancer. Acceptable levels were lowered from 50 *μ*g/L to 10 *μ*g/L in drinking water by the WHO [4]. Arsenic-containing compounds are applied in plant pesticides and insecticides and arsenic environmental contamination represents a global health problem, particularly from leaching into ground water. When the soluble levels exceed 50 *μ*g/L in drinking water as in many regions of Bangladesh, arsenic becomes a particular health concern as it has recently been associated with increased cancer rates appearing after consumption with a lag time up to 10–20 years [5].

Whilst the carcinogenic aspects of arsenic compounds are not the focus of this review, nevertheless this undesirable aspect needs to be raised. Thus, arsenic and its methylated species are known carcinogens but this description is probably inaccurate as they act more as cocarcinogens by facilitating/promoting the induction of tumors of the skin, urinary bladder, and lung rather than directly inducing cell transformation and oncogenesis [2, 6]. As cocarcinogens, mechanisms include indirect effects such as DNA damaging genotoxicity by altered DNA methylation as well as inducing high levels of oxidative stress leading to altered cell proliferation and tumor promotion (detailed in [7]).

In terms of chronic toxicity, the 50% lethal dose of arsenic as arsenic trioxide in mice received by the oral route varies from 15–48 mg/kg, whereas the acute lethal dose in humans varies from 1–3 mg/kg body weight [4]. Despite its toxicity, arsenic has been applied in Chinese medicines for thousands of years as refined preparations of realgar (As_4_S_4_) or orpiment (As_2_S_3_) [8] which are two rare mineralised forms. Realgar, easily discerned by its bright red and orpiment with a browny yellow appearance are ores often occurring close to each other in hydrothermal veins of precious metal sulfide ores or hot spring deposits as volcanic sublimate products (crystallized from gases). Hence, arsenic is a common waste product from the mining of metal ores and the name realgar is possibly derived from Arabic words for “powder of the mine” (rahj al ghar).

The long history of medical applications has included treating many diseases such as cancers administering micromolar levels in patients and, in particular, arsenic compounds have proven to be very effective against certain hematological malignancies (reviewed in [9, 10]). Thus, arsenic oxides and derivatives have been established as effective treatments for acute promyelocytic leukemia (APL), and they are being tested as therapies for a range of other hematological cancers including myelodysplastic syndromes, multiple myeloma, and chronic myelogenous leukemia (CML). The results of clinical trails using arsenic-based drugs in cancer therapy have been extensively reviewed [8–12]. Hence, this review mainly concerns those factors that explain the antineoplastic mechanism of action of arsenic-based drugs, distinguishing their selectivity for killing certain types of cancer cells from their potential for toxicity to normal cells in the body. The reason for their efficacy and selectivity in treating certain hematological malignancies is covered in the last section of this review. However, the mechanisms of action of arsenic-basedcompounds on cells must first be described in detail to provide sufficient understanding to enable their selective targeting of specific types of cancer cells to be duly evaluated.

Water distributes the majority of inorganic arsenic in the biosphere either as pentavalent arsenate (As^5+^, As(V)) or trivalent arsenic (As^3+^, As(III)) [2]. In solution, the pH and redox conditions affect the chemical state of arsenic that predominates. Thus, in highly oxidised environments, the arsenate (As^5+^) form predominates as one of four major species of arsenic acid; H_3_AsO_4_, H_2_AsO_4_^−^, HAsO_4_^2−^, and AsO_4_^3−^ [13]. However, mildly reducing conditions such as those generally present inside all mammalian cells [14] will favour the reduction of the pentavalent arsenate (As^5+^) to the trivalent arsenic (As^3+^). Arsenous acid with the formula As(OH)_3_ results after the slow hydrolysis of arsenic trioxide in water. As the pH increases, arsenous acid is converted to the arsenic oxide ions [AsO(OH)_2_]^−^, [AsO_2_(OH)]^−2^, and [AsO_3_]^−3^ [13].

## 2. METHYLATED FORMS OF ARSENIC SHOW INCREASED CYTOTOXICITY INSIDE CELLS

Arsenic combines readily with many elements; and bacteria have evolved systems to detoxify inorganic arsenic to form organic arsenic-containing compounds such as methylated forms. Plant and insect pesticides have been made using organic arsenic derivatives; and monomethylarsonic acid (MMA(V)) and dimethylarsinic acid (DMA(V)) are used in products for weed control. Also, MMA(V) and DMA(V) are metabolites of inorganic arsenic, formed intracellularly in mammals, primarily in the liver. The metabolic process of inorganic arsenic conversion is known as biotransformation and appears to enhance the excretion of arsenic from the body, involving formation of methylated compounds of trivalent arsenic as intermediates. The metabolism involves reduction to a trivalent state and oxidative methylation to a pentavalent state. In addition, reductases present in cells and other reactions facilitate the reduction of arsenic acid [As(V)] to the arsenous [As(III)] form including methylation of the arsenous form, mainly via the liver, to produce mono-, di-, and trimethylated species [2]. The main enzyme involved in methylation of As(III) is arsenic methyltransferase (As3MT) that requires glutathione (GSH) to promote the reaction and a cycling redox system such as thioredoxin (reviewed in [15]) to detoxify arsenic-containing compounds. Many animal species convert inorganic arsenic-containing compounds into mono-, di- and tri-methylated arsenic species which are mostly then secreted in the urine [16]. This is because the methylated forms are not as well absorbed by cells compared to the inorganic forms, although they have much greater cytotoxicity if they do enter cells [15, 17].

The reductive metabolism of arsenic has an important role in its toxicity. The trivalent arsenic-containing compounds, including the methylated organic forms have much greater potency than the pentavalent arsenic-containing compounds as cytotoxics and carcinogens [18–23]. For example, in cytotoxicity assays, the IC50 values for cultures of primary hepatocytes, keratinocytes, and epithelial cells ranged from 3 *μ*M to well over 20 *μ*M for trihydroxidoarsenic salts whereas for monomethylarsonous acid [MMA(III)], the values were consistently much lower at only several *μ*M for the equivalent normal cell types [24–26]. Organic arsenic-based ingredients are commonly used as feed additives in poultry farming to increase weight gain by preventing bacterial and parasitic infections, thereby increasing feed efficiency and improving pigmentation. The three major arsenic-containing compounds used in this manner are arsenilic acid (p-aminophenyl arsonic acid), roxarsone (4-hydroxy-3-nitrophenylarsonic acid), and nitarsone (4-nitro-phenylarsonic acid) [22, 23]. The metabolism of these arsenic-containing compounds and waste products produced by birds and mammals consuming them is still uncertain at present and what the global environmental impact might be.

## 3. CELLULAR ACTIONS OF ARSENIC-CONTAINING COMPOUNDS

### 3.1. Inhibitors of energy metabolism: effects on glycolysis and oxidative phosphorylation

At the biochemical level, inorganic arsenic in the pentavalent state (As(V), arsenate, AsO_4_) resembles a phosphate (PO_4_) group in structure and it can replace phosphate in many reactions. The mitochondria is a major intracellular site where arsenate is metabolised, taken up As(V), rapidly reducing it, and exporting the As(III) product back into the cytosol [27]. The specific location of the site(s) for arsenic reduction in the mitochondria has not yet been defined. However, arsenate can affect oxidative phosphorylation by binding to the Fo/F1 ATP synthase [28]. Arsenate can be used by the ATP synthase more efficiently than phosphate depending on the Ca2+ levels [29], producing ADP-arsenate which unlike ATP becomes rapidly hydrolysed and unable to form stable high-energy compounds [30]. It is suggested that the structure and charge similarities of PO_4_^3−^, AsO_4_^3−^, and SO_4_^2−^ result in indiscriminate binding to at least two sites located in the mitochondrial matrix [31]. At one site, occupation by any of these three anions results in protection against uncoupling of the mitochondrial proton gradient by K^+^; at the second site, in the Fo/F1 ATP synthase, AsO_4_^3−^, and SO_4_^2−^ compete for binding against PO_4_^3−^, leading to the inhibition of ATP production [31].

Intriguingly, As_2_O_3_, whilst shown to have no effect on oxidative phosphorylation levels in HeLa [10 *μ*M] and AS-30D [100 *μ*M] hepatoma cancer cells, significantly inhibited glycolysis, particularly during the exponential growth phase of these cells when they were actively respiring and producing 70% of their ATP from mitochondria [32]. Several other studies have also shown that arsenic compounds do not affect oxidative phosphorylation. Thus, with submitochondrial particles in the presence of an ATP regenerating system, 20 mM arsenate had no effect on NADH formation, ATP hydrolysis, and Pi ↔ H20 exchange [33]. In a related study with isolated liver mitochondria, As_2_O_3_ was again shown to have no effect on oxygen consumption, or the respiratory control ratio at a concentration (10 *μ*M) found to be maximally effective in promoting apoptosis in whole cells [34]. However, at higher concentrations (> 50 *μ*M), pyruvate/malate-supported respiration (via complex I) became blocked, but there was no effect on either complex II or IV. The inhibitory effect on complex I was reversed by the addition of the reducing agent, dithiothreitol, indicating that direct oxidative damage was involved. In addition, it was shown that even with the block in complex I, the cells continued to maintain cellular ATP levels through glycolysis, and hence, depletion of cellular ATP was not the cause for the cytotoxicity of As_2_O_3_ [34]. Probably the most definitive evidence for the importance of mitochondria in mediating As_2_O_3_ killing of cancer cells comes from studying cells lacking mitochondrial function [35]. A subclone of mitochondrial respiration deficient cells was derived from the HL-60 human leukaemia cell line by growth in the presence of ethidium bromide to mutate the mitochondrial genome, and these cells are known as HL-60 “*ρ* ^0^” cells [35]. Due to the lack of mitochondrial respiration, *ρ* ^0^cells depend on glycolysis for their energy source and, as would be expected, produced substantially less superoxide radicals (*∼*20% of the control cells). When these *ρ* ^0^ cells were incubated with *∼*10 *μ*M As_2_O_3_, they were resistant to the drug, revealing that mitochondrial respiratory function is required for the cytotoxic actions of As_2_O_3_ [35].

The inhibition of glycolysis by arsenic-based drugs appears unlikely to be a significant factor involved in the drug-induced killing of cancer cells. Thus, incubating cells in glucose-deficient medium to block glycolysis had no significant effect on the As_2_O_3_^−^ [30 *μ*M] mediated levels of cell death in the Jurkat cell line [36]. However, when glycolysis was blocked and mitochondrial respiration inhibited using oligomycin A, the cells became very sensitive to As_2_O_3−_ mediated cell death. In this regard, it is also worth noting that studies of individual glycolytic enzymes analysed in purified form in vitro have shown arsenite and arsenate to be relatively weak inhibitors. Hence, for hexokinase (IC50: 15mM for arsenate [37, 38]), phosphofructokinase (IC50 > 5 mM for arsenite; [39]) and pyruvate dehydrogenase (PDH IC50: 80–120 *μ*M arsenite; [40]), relatively large concentrations were required to inhibit these enzymes. In fact, arsenate has been shown to stimulate the activities of the two important glycolytic enzymes, hexokinase [37] and GAPDH [41], by overcoming product inhibition in these reactions. In the case of GAPDH, arsenate acts catalytically to promote the oxidation of phosphoglycerate [42] and the reaction involved the formation of an arsenate analogue of the phosphate ester as an intermediate which rapidly hydrolysed, helping to drive the reaction forward [33, 43]. This process has been commonly described in relation to the effects of arsenate on numerous enzymatic reactions involving phosphate and has been termed “arsenolysis” [33].

Given the relative insensitivity to the direct effects of arsenic compounds shown by the glycolytic pathway, it follows that it is unlikely that glycolytic inhibition results from direct binding and modification of the enzymes in this pathway by arsenic-based drugs. More likely, the inhibition of glycolysis results from an indirect effect, caused by the actions of arsenic compounds in modifying mitochondrial respiration leading to production of ROS which then acts to inhibit the glycolytic enzymes. Additional support for the mitochondrial-mediated ROS involvement in the action of arsenic to inhibit glycolysis comes from studies where As_2_O_3_ was found to be *∼*38 times more potent in cells than in the pure preparation at inhibiting pyruvate dehydrogenase [40]. Also, inhibiting mitochondrial respiration suppressed the resulting inhibition of pyruvate dehydrogenase activity and H_2_O_2_ production by this drug. Furthermore, the inhibition of pyruvate dehydrogenase by As_2_O_3_ was shown to require the Fenton reaction occurring via hydroxyl radical intermediates [40]. The mitochondrial effects of arsenic compounds are detailed later in this review and to reiterate at this point, the evidence indicates that the actions of arsenic compounds on glycolysis are not the main cause for the cytotoxic effects of these drugs at clinically relevant concentrations (1–6 *μ*M) required in plasma for the killing of cancer cells in APL patients [8, 44].

### 3.2. Interconversion of As(III) ↔ As(V) in cells

Under conditions of high mitochondrial respiration inside cells, it is possible that trivalent arsenicals inducing significant production of ROS as superoxide, peroxide, and hydroxyl radicals can also result in oxidation to produce arsenate (AsO_4_^3−^) ionic species [45, 46]. Consequently, the impact that arsenic compounds will have on any given cell will most likely depend on the state of cellular respiration and production of ROS affecting the arsenic speciation and whether the cell is dependant on glycolysis versus mitochondrial respiration for its ATP synthesis. Glyceraldehyde-3-phosphate dehydrogenase (GAPDH), the glycolytic enzyme abundantly found in all cells and especially blood cells and liver, is a major intracellular arsenate reductase [47] requiring GSH, NAD, and glycolytic substrate [48, 49]. Given that the levels and specific activity of GAPDH is much higher in malignant cells than in normal cells [50], this could contribute to the rapid reduction of As(V) species in their cytosol into more toxic As(III) forms.

In the GAPDH reaction, As(V) reduction may take place during, or as a consequence of the arsenolytic cleavage of the thioester bond formed between the enzyme's Cys149 residue and the 3-phosphoglyceroyl moiety of the substrate. Hydrolysis of 1-arseno-3-phosphoglycerate is at least 2000 times faster than hydrolysis of the normal substrate 1,3-diphosphoglycerate under the same conditions [51]. Hence, GAPDH is proposed as one of the key cellular converting enzymes for reducing As(V) to As(III). Although purine nucleoside phosphorylase was proposed to be an arsenate reductase [52], this was later refuted [53]. The other major class of As(V) reductases in cells are the glutathione S transferases and of these, the omega form or GSTO1 appears to be most important. Thus, GSTO1 can reduce arsenate to arsenite, MMA(V) to MMA(III), and DMA(V) to DMA(III) and deletion of the GSTO1 gene in mice reduced the extent of biotransformation by 30–80% in most tissues examined [54].

### 3.3. Structure and reactivity of arsenic-containing compounds with reduced thiols

Arsenic has a high affinity for sulfur and hence, reactive sulfur-containing molecules such as reduced thiols with an available sulfur atom have a significant propensity for binding to arsenic [55, 56]. As(III)-containing compounds exist as trigonal pyramidal structures and this is also the structure formed upon binding of arsenic ions to cellular proteins in vivo where the sulphur atoms of thiolate groups act as coordinating ligands. The resulting arsenic-thiol linkages are mainly responsible for the ability of arsenic to modulate the function of various key molecules, enzymes, and ion transporters inside cells and this intracellular action of arsenic is discussed in detail in this section. Arsenic-containing compounds react with mono- and dithiols, particularly the latter when two thiols are located in close proximity, acting to cross-link the thiols together.

Some debate exists about the structures and speciation of arsenic-containing compounds (both inorganic and organic forms) in solution. In the absence of sulfide, As(III) hydroxide complexes are the major arsenic-containing species and these structures probably adopt a trigonal pyramidal structure with the arsenic atom at the apex [57–59]. This trigonal pyramidal structure provides the potential for As(III) to coordinate linkages with several proximal thiol groups. This is believed to be the case in bacterial enzyme systems such as the ArsR repressor protein where it is likely to bind three Cys atoms [60]. Thus, in the more toxic form as the trivalent state (As(III)) inorganic and organic (methylated) arsenic reacts with critical thiols in proteins, inhibiting their function, as is the case with the bacterial ArsR protein. For example, As(III) was shown to target the reactive sulfhydryl group at the active site of thiolase(s) involved in ketogenesis from acetyl Coenzyme A [61].

The pentavalent species of inorganic arsenate (AsO_4_^3−^) favoured to exist in oxidised environments, as well as organic forms of As(V) have a different structure with a trigonal-bipyramidal shape where the As(V) atom is located at the centre, co-ordinating to three equatorial and two polar atoms [62, 63]. In comparing the two different structural states of As(III) and As(V), it is not yet clear why the trivalent methylarsenic-containing compounds show a much higher toxicity than their pentavalent analogues [24, 64]. However, it could relate to cellular uptake as trivalent organoarsenic compounds are more membrane permeable than the pentavalent species [65]. Also, trivalent arsenic bonded at a phenyl ring is able to form much more stable covalent cross-links to cysteine residues compared to arsenic in small molecules such as arsenious acid or arsenite [66]. Furthermore, the organic trivalent arsenic-containing compound, phenylarsine oxide [0.1–0.5 *μ*M], is much more potent than the simple arsenite [1–10 *μ*M] in its cytotoxic activity in APL cells [67].

One major drawback with phenylarsine oxide as a potential cancer therapy is its high toxicity in vivo and its nonselectivity for cancer versus normal cells, resulting in cytotoxicity in normal endothelial cells in the same concentration range (0.2 *μ*M) [68]. Thus, phenylarsine oxide, is precluded from application in clinical cancer therapy, without being further modified as a drug. The greater reactivity of phenylarsene oxide and associated cytotoxicity is in agreement with the results outlined above [66] where mass spectrometric analysis of different complexes of peptides and proteins with arsenic-containing species revealed that inorganic arsenite or arsenates did not interact well with cysteine or glutathione, whereas the organic phenylarsine (3+) oxide did. In addition, three different phenyl arsenic acids and dimethylarsinic acid that all contained As(V) also formed complexes with glutathione [66]. Hence, the bulky hydrophobic groups with electron withdrawing *π* orbitals (in the case of phenyl groups) may promote more stable bonds between the arsenic atom and sulfur groups inside cells, modulating a larger range of enzymes and proteins with important functions for maintaining cell viability.

In cells, the most common reactive species that are available for interaction with arsenic are the abundant free thiol moieties in the tripeptide glutathione (*γ*Glu-Cys-Gly, GSH) and the free amino acid, cysteine. Thus, arsenic-based drugs can react by coordinating binding to free (reduced) thiol groups such as those on cysteine, particularly those of thioredoxin and glutathione as the major intracellular thiol species important in cellular redox regulation. It was observed early on that arsenite and phenylarsine oxide in particular, but not arsenate, reacted with vicinal thiol groups on proteins [69, 70]. Since demonstrating that phenylarsine oxide was particularly effective at cross-linking vicinal thiols in the active site of tyrosine phosphatases [71], the range of proteins -containing vicinal cysteine residues with which phenylarsine oxide reacts is increasing. Recent examples include the small GTP binding Rho protein family [72] and the mitochondrial carnitine/acylcarnitine transporter [73]. Phenylarsine oxide, as a strong inhibitor of tyrosine phosphatases would increase tyrosine phosphorylation levels of enzymes in cellular growth signalling pathways. Whether the inhibition of tyrosine phosphatases and other enzymes contributes to the toxic effects of arsenic-containing compounds in normal cells is not clear, but is likely an important contributing factor to its general cytotoxicity making phenylarsine oxide unsuitable for cancer therapy.

Analysis of the interactions of As(III) with glutathione or cysteine in vitro in aqueous solutions by equilibrium binding and use of biophysical techniques including NMR, electronic spectroscopy, and potentiometry revealed that As(III) binds either of glutathione or cysteine with similar equilibrium constants [74]. However, several analytical studies by mass spectroscopy have revealed that arsenate and arsenite do not complex readily with glutathione or cysteine, but prefer to react with the thiols on reduced thioredoxin molecules [reviewed in [66]]. This was confirmed by analysis of more biologically relevant samples from the intracellular environment of HeLa cells where cytosolic thioredoxin 1 (TRX1) and, in particular thioredoxin 2 (TRX2) in the mitochondria, was shown to be highly reactive with arsenite (10 *μ*M) whereas little reactivity was detected with cellular GSH/GSSG [75].

Studies with thioredoxin reductase (TxR) purified from mouse liver showed that arsenic-containing compounds with As(III) and arsinothiols (complexes of As(III) with GSH or L-cysteine) were extremely potent inhibitors of this enzyme [24]. Methylarsenic(III)oxide was most potent with a Ki*∼*100 nM, as an irreversible competitive inhibitor. The effects on purified glutathione reductase (GR) showed that the levels of inhibition were not as marked with inorganic As(III) and As(V) oxides showing IC50s in the 10–50 mM range, whereas for methylarsinic(III)oxide it was *∼*9 *μ*M. Studies on this enzyme in whole cells as hepatocytes showed the IC50 to be reduced to *∼*3 *μ*M for methylarsinic(III) oxide and for As_2_O_3_ > 100 *μ*M [24]. Hence, these observations strongly support a role for the components thioredoxins and the thioredoxin reductase system as providing cellular targets that are very sensitive to inhibition by arsenic-based drugs in the low micromolar range. It also explains the importance of studying the chemistry of arsenic-containing compounds in the context of both purified enzymes as well as whole cells as biological systems in order to obtain meaningful results. Thus, although the GSH/glutathione transferase system undoubtedly plays an important role in arsenic sensitivity of cells (see below in Section 4 for details), it would appear that the thioredoxin system might represent the most immediate point of sensitive reactivity in relation to cytotoxicity.

### 3.4. The major mechanism of action of arsenic-containing compounds: modifying mitochondrial function and redox regulation of the production of reactive oxygen species

**Figure 1:**
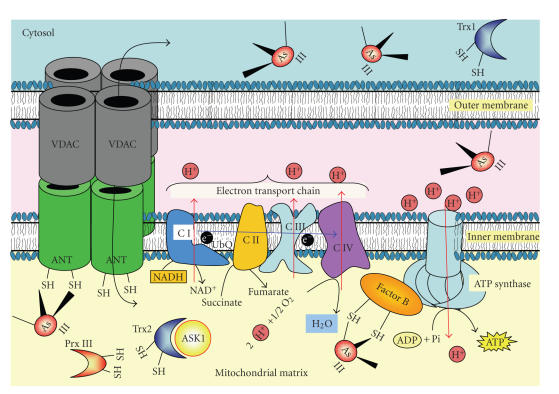
*Many vicinal thiol-containing redox proteins are major mitochondrial targets for binding of arsenic-containing compounds.* Arsenite and particularly organic arsenites will disrupt the normal redox systems functioning in the mitochondrial matrix and intermembrane space by targeting vicinal thiols in proteins and enzymes that regulate these systems. Such enzymes include Peroxiredoxin III (Prx III), Thioredoxin 2 (Trx2). In addition, several key mitochondrial functions are affected, including Factor B regulating the ATP synthetase activity, the adenine nucleotide transporter, ANT, amongst others. See text for further detail.

Mitochondria are a main source of ROS in cells (reviewed in [76]). Thioredoxin (TRX), NADPH, and thioredoxin reductase (TxR) comprise the thioredoxin system that has multiple functions in cells including in redox signalling via interactions with other proteins, in transcriptional regulation, control of the reduced intracellular redox environment, cell growth, defense against oxidative stress and control of apoptosis (reviewed in [77]). As outlined in the previous section, the thioredoxin system is very sensitive to arsenic-based drugs and may well be the basis for one of the important mechanisms for their actions in inducing cancer cell death. The TRX system operates as a thiol-disulfide exchange reaction (see Figure 1). TRX1 and TRX2 are key regulatory isozymes that catalyse the reduction of protein disulfide bonds. They are cofactors of the apoptosis signal-regulating kinase 1 (ASK1) that mediates TNF cytokine and oxidative stress-induced apoptosis via the mitochondrial dependent pathway [78].

In their reduced forms, cytosolic TRX1 and mitochondrial TRX2 each contain two vicinal thiol groups in their active site sequence as –C–G–P–C–. TRX 1 in the cytosol and TRX2 in mitochondria bind to Cys 250 and Cys 30, respectively, in the regulatory N-terminal domain of ASK1 and can cooperatively maintain the enzyme in the inhibited state. However, activation by TNF resulting in increased production of ROS and leads to the oxidation of the TRX dithiol group to a disulfide. Under these conditions, the thioredoxins no longer bind to ASK1 and loss of TRX2 binding to mitochondrial-located ASK1 can lead to apoptosis in a JNK-independent manner, whereas cytosolic ASK1 upon loss of TRX1 binding then becomes activated as a MAPKKK resulting in JNK activation, Bid cleavage and Bax translocation to the mitochondria [78]. Since it is known that the thioredoxins are major targets of arsenic-containing compounds (see above and [75]), it can be predicted that arsenite-mediated oxidative binding to thioredoxins will induce a similar outcome as TNF signalling, leading to the release and activation of ASK1 and induction of apoptosis.

Another thioredoxin-associated protein of importance in thiol-mediated redox regulation in mitochondria is thioredoxin peroxidase II (TPX-II, also known as peroxiredoxin III, Prx-III), an enzyme abundantly expressed in the mitochondria of cancer cells that protects the cells from oxidative stress [79, 80]. PrxIII is an important antioxidant that acts in conjunction with TRX2/TXR (Figure 1) in the mitochondria to remove peroxides such as H_2_O_2_ and offset the apoptosis inducing effects of increased levels of H_2_O_2_. However, PrxIII contains three Cys residues, two of which are involved as redox-active sites in the formation of a stable intersubunit disulfide-bonded dimer, which is then reduced by thioredoxin to the monomer. PrxIII was a more abundantly expressed arsenic-binding protein when comparing arsenic resistant cells to normal cells by phenylarsine oxide affinity chromatography [81]. Hence, PrxIII is very likely to be another protein whose function is inhibited by arsenic-containing compounds leading to the promotion of apoptosis.

**Figure 2:**
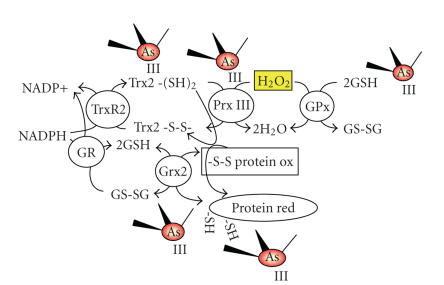
* Mitochondrial redox systems regulating ROS levels via thiol-disulfide exchange/coupling reactions.* The mitochondrial form of Thioredoxin (Trx2) is likely to play the major role in reducing disulfides formed by vicinal thiols in both the mitochondrial Peroxiredoxin III (Prx III) and other proteins. Prx III is one of the main ways by which cancer cells can reduce their levels of H_2_O_2_ built up during active respiration. The glutathione redox system comprising GSH/GSSG, glutathione reductase, glutaredoxin, and glutathione peroxidase, although present in the mitochondria, is more likely to only become of major importance during the more extreme conditions of oxidative stress. Both of these systems are targets for inhibition by arsenic-containing compounds. See text for further detail.

Increasingly, it is becoming apparent that dithiols-containing redox proteins, particularly those present in the mitochondria, act as controlling sensors during responses to changes in cellular redox. Many thiol redox proteins contain a vicinal pair or more of reactive thiol groups [82, 83] capable of binding with arsenic-containing compounds in a similar manner to those in the thioredoxin system. The most important of these are located in the mitochondria, a point of extreme sensitivity to arsenic-containing compounds whose actions culminate in triggering the apoptotic pathways via the induction of reactive oxygen species, leading to the killing of the cancer cells (Figures 1, 2). For example, two members of the glutaredoxin (GRX) family, including GRX2 located primarily in the mitochondria, catalyse GSH-dependent TRX-disulfide redox and protein thiol-disulfide redox reactions, particularly reversible glutathionylation of protein sulfhydryl groups [84]. Human Grx1 and Grx2 contain C–P–Y–C and C–S–Y–C active sites ; have three and two additional structural Cys residues, respectively; and are therefore likely to react with arsenic-containing compounds, although no reports of this could be found.

Another mitochondrial protein targeted by arsenic-containing compounds with a vicinal dithiol group is regulatory protein “Factor B.” Addition of recombinant factor B back to bovine submitochondrial particles depleted of this protein restored energy coupling activity. Thus, reverse electron transfer from succinate to complex I enabling NAD+ reduction, electron transport chain function and oxidative phosphorylation/^32^Pi-ATP exchange of the ATP synthetase complex were reactivated [85] as was increased exchange activity of complex V [86]. Thus, the F0-F1 ATPase activity requires Factor B coupled to it for full activation. However, factor B contains six thiols and Cys 92 and Cys 94 in the bovine form were shown to bind phenylarsine oxide [87] and phenylarsine oxide or arsenite inhibit factor B coupling activity [88]. From all of these studies, it is becoming clear that redox changes to vicinal thiols affect the regulation of mitochondrial function and that these thiols are also major targets for inhibition by the arsenic-containing compounds (Figures 1, 2).

## 4. THE ADENINE NUCLEOTIDE TRANSPORTER (ANT): A CRITICAL TARGET OF ARSENIC-CONTAINING COMPOUNDS IN THE MITOCHONDRIA

A channel formed by the association of two proteins, the voltage dependent anion channel (VDAC) in the outer mitochondrial membrane and the adenine nucleotide transporter (ANT) in the inner mitochondrial membrane (Figure 1), is a complex involved in the induction of apoptosis activated via the mitochondrial pathway (see [89], Figure 3). The two components of this complex form part of the mitochondrial permeability transition pore (MPTP), a megachannel mediating release of molecules from the mitochondria activating apoptosis. A rapidly increased permeability of the inner mitochondrial membrane (mitochondrial permeability transition) leads to apoptosis that is mediated by the MPTP.

Arsenite induces apoptosis by a direct effect on the MPTP [90, 91] and VDAC has been shown to play an essential role in opening of the permeability pore and cytochrome c release induced by arsenic trioxide, which also caused VDAC to homodimerise [92]. In addition, the thiol-reactive compound 4,4’-diisothiocyanostilbene-2,2’-disulfonate (DIDS) has been shown to block the VDAC channel [93] and inhibit ROS-mediated cytochrome c release by VDAC [94]. Hence, the data indicates that VDAC contains critical Cys residues that can undergo intermolecular cross-links mediated by reaction with arsenic.

Analysis of ion channel activity of purified ANT-containing lipid bilayers also revealed that As2O3 treatment [30 *μ*M] altered the ANT channel electrophysiological properties [36]. Interestingly, glutathione depletion leading to increased ROS may play an important role in the action of arsenic trioxide [91]. Whereas in both normal and cancer cells, glutathione S transferase (GST) is found to interact with ANT, during the induction phase of apoptosis, GST dissociates from ANT suggesting that GST/GSH may act as a repressor of MPTP and ANT pore opening [91, 95]. This is supported by the observation that increasing the expression of the enzyme GSTP1 in Jurkat and Raji leukemic cells renders them more resistant to arsenic trioxide-induced apoptosis at clinically relevant levels [1-2 *μ*M]. GSTP1 expression in these cells is also accompanied by accumulation of lower levels of H_2_O_2_ production [95].

Both of the mammalian proteins, VDAC and ANT, contain two or more Cys residues in their structure thereby providing reactive thiol groups whose modification affects their function. It has been established that the redox state of thiol reactive groups are important for activation of the mitochondrial permeability transition [94, 96, 97]. Consequently, thiol cross-linkers such as DIDS, diamide, and phenylarsine oxide [20–100 *μ*M] affect VDAC and ANT channel function and activation of mitochondrial permeability transition [93, 94, 96–98]. Many published results, such as cross-linking experiments, protein/inhibitor stoichiometry, chimeric dimers, analytical ultracentrifugation, or neutron scattering, indicate that the ANT carrier acts as a dimer and this or higher oligomers are involved in membrane permeability transition [99, 100].

Facing out into the mitochondrial matrix, ANT has three exposed loop regions containing a conserved repeat structure with one Cys residue in each loop. These Cys residues are important to the process of ANT dimerisation, but it is not clear how this operates and whether the Cys residues form intermolecular disulfide bonds or not [101]. Nevertheless, copper-o-phenanthroline is able to dimerise ANT by intermolecular cross-linking of Cys 56 (in the first matrix loop) [98]. In addition, phenylarsine oxide, eosin 5-maleimide, and diamide form intramolecular cross-links between Cys 160 and Cys 257 on the other two matrix loops, restricting ANT in the open conformation, promoting mitochondrial permeability transition [98]. Arsenic trioxide is much weaker than phenylarsine oxide at binding to the ANT Cys residues [96, 97] and this may explain the greater sensitivity exhibited by APL cells to phenylarsine oxide [IC50: 0.1 *μ*M] than to As_2_O_3_ [IC50: 4 *μ*M] [67].

Single thiol interacting compounds such as N-ethylmaleimide (NEM) can inhibit the mitochondrial permeability transition and this could be either the result of direct interaction with the key Cys residues on the matrix loops of ANT or indirectly via reaction with GSH and thereby preventing GSH from being oxidized and catalysing disulfide bridging between the adjacent thiol groups in the ANT loops [98]. NEM or monobromobimane, in the 25–50 *μ*M range, preferentially react with GSH, leading to its modification in mitochondria and thereby prevents GSH from being oxidised. As a result, NEM inhibits mitochondrial permeability transition activation by the thiol reactive compounds, diamide or t-butylhydroperoxide, implying a role for GSSG in the action of these agents on the permeability transition [96, 97]. Arsenites, albeit that much higher concentrations would be required given their lower affinity for glutathione interaction, could have a similar action. Thus, high levels of arsenites could modify and inhibit glutathione redox control such that glutathione-based enzymes are unable to function, as well as directly mediating disulfide cross-linking of ANT, leading to increases in cellular ROS production, MPTP, and apoptosis. However, given their low reactivity with glutathione systems, this appears to be unlikely as opposed to the indirect action via the mitochondrial effects leading to increased ROS production which then reduces cellular GSH levels.

The multidrug resistance (MDR) protein MRP1/ABCC1 has been shown to transport AsIII out of cells as a tri-GSH conjugate (As-(GSH)3), and glutathione S-transferase (GST) probably facilitates the process [102]. This is likely to be part of the normal cell and cancer cell resistance mechanisms against the cytotoxic effects of arsenic-based compounds. GSH-depleted cells are more sensitive to killing by arsenic-containing compounds [103] and transfection of cells to express glutathione S transferase protects them from arsenite inducing death by promoting arsenite transport from the cells and decreasing ROS levels [104, 105]. In support of this proposal, the long-term exposure of cells to arsenic-containing compounds induced increased expression of glutathione S transferase and MRPs [106]. Arsenic levels do not attain very high levels in blood plasma of patients, rapidly becoming eliminated [8, 44, 107]. This removal probably results from efficient uptake of As-(GSH)3 via MRP2 in the proximal tubules of the kidneys as part of the detoxification process during the excretion of arsenic-based drugs predominantly into the urine [108, 109]. The remainder is mostly removed via uptake in the liver and secretion as bile [110–112]. As-(GSH)3 may be an important part of the metabolic process for converting inorganic As(III) to methylated species during detoxification in the liver by ASMT1/Cyt19 [113].

## 5. MODIFIED ORGANIC ARSENIC STRUCTURES WITH INCREASED POTENCY AS ANTINEOPLASTIC AGENTS

Arsenic-containing compounds substituted with organic groups such as modified phenylarsine oxides have been synthesized and examined for their cytotoxic effect on human leukemic cells and breast cancer cells in culture. Some of these compounds were found to exhibit potent cytotoxic anticancer activity, particularly against human breast cancer and leukemic cell lines, including primary leukemia cells, at micromolar concentrations. One of these compounds, the novel glutathionyl peptide trivalent arsenic-containing compound para 4-[*N*-(*S*-glutathionylacetyl)amino]phenylarsenoxide (p-GSAO) shows promise as a novel antineoplastic drug and is now in clinical trials. P-GSAO, like phenylarsine oxide, inactivates ANT-mediated ATP/ADP transport and triggers Ca^2+^-dependent MPTP opening by cross-linking the critical Cys residues of ANT. This leads to increased production of cellular ROS, ATP depletion, mitochondrial depolarization, and apoptosis of angiogenic endothelial cells and inhibition of tumor growth in mice with no apparent toxicity [114]. However, the action of p-GSAO was indirect, and did not appear to be as a result of selective tumor cell toxicity. Rather, p-GSAO inhibited the proliferating, but not growth-quiescent endothelial cells in vitro and angiogenesis in vivo and thus acted to eliminate tumors by blocking their blood supply [114, 115]. The trivalent arsenic-containing moiety of p-GSAO was shown to cross-link the matrix facing Cys160 and Cys257 thiols of ANT [114] and effectively locks ANT into the open configuration. Inactivation of ANT by p-GSAO causes an increase in superoxide levels, proliferation arrest, ATP depletion, mitochondrial depolarization, and apoptosis in the dividing endothelial cells.

It is likely that the arsenic-containing moiety of p-GSAO reacts similarly as does arsenite (see above) with one or two molecules of glutathione before it is removed from the cell by MRPs [116]. Tumor cells export p-GSAO much more efficiently than endothelial cells because they have higher MRP1 or MRP2 activity and cellular glutathione levels [116] and this may explain why p-GSAO is not highly effective at inhibiting tumor cell growth in vivo. In addition, the greater water soluble properties of p-GSAO than other arsenic-containing compounds, particularly organic species should help to retain p-GSAO in the intravascular system where it is more likely to affect endothelial cells and inhibit tumor angiogenesis.

Interestingly, although the para form of GSAO revealed no apparent toxicity in treated animals and inhibited tumor growth leading to phase I clinical trials as an anticancer agent [114], the ortho form (o-GSAO) was toxic and this was proposed to result from increased accumulation of the drug in cells, including normal cells due to loss of multidrug resistance efflux [116]. Consequently, o-GSAO is unlikely to be of much further interest as an antineoplastic agent, whereas the clinical efficacy of p-GSAO is eagerly awaited.

## 6. TARGETING OF CANCER CELLS: SELECTIVE UPTAKE AND DELIVERY INTO SPECIFIC TYPES OF CANCER CELLS

As(III) as the anhydrous form of As(OH)_3_ (Trisenox, Cell Therapeutics, Seattle, Wash, USA) received FDA approval in 2000 as a chemotherapeutic agent for the treatment of APL [117]. Acute promyelocytic leukemia (APL) is associated with reciprocal and balanced chromosomal translocations always involving the retinoic acid receptor alpha (RARalpha) gene on chromosome 17 and variable partner genes on distinct chromosomes. RARalpha fuses to the promyeloctyic leukemia (PML) gene in the majority of APL cases (reviewed in [118]). Arsenic trioxide is particularly effective at killing APL cells and this was proposed to be the direct result of its ability to induce the relocalization and degradation of the nuclear body protein PML, as well as the degradation of PML-RARalpha in APL cells [119–122]. However, this seems unlikely to be the main mechanism of action for arsenic trioxide given that no differences in sensitivity to growth inhibition and killing by apoptosis have been observed between wild-type and PML^−/−^ cells [123].

Arsenic trioxide as a single agent has provided 86% complete hematologic remission with minimal toxicity in APL patients [124], equal to any of the current standards of care for treating APL, including the combination of all-trans retinoic acid (ATRA) plus chemotherapy [125]. This raises the question why APL cells are very sensitive to arsenic-containing compounds like arsenic trioxide. It would appear that the reason is because APL cells express the transmembrane transporter protein, aquaglyceroporin 9 (AQP9) involved in arsenic uptake [126] at much higher levels in APL cells than in other leukemic cell types and that correlates with arsenite sensitivity [127]. In this regard, it is worth noting that aquaglyceroporins AQP7 and AQP9 are present in normal cell types. Interestingly, AQP9 is primarily expressed in human lung, liver, and leukocytes [128] and this may help explain arsenic toxicity, given that liver is one of the main organs affected. The fact that AQP9 provides APL cancer cell specificity with high response rates suggests that if arsenic-containing compounds could be targeted for specific delivery into cancer cells, then they would represent outstanding agents for killing these cells. However, further modifications will be required to provide suitable drug targeting for improved delivery of arsenic-containing compounds to cancer cells.

## 7. CONCLUSIONS

Vicinal thiols located in key enzymes and proteins provide targets for reaction with arsenic-containing compounds, particularly organic derivatives such as phenylarsenic-containing compounds that favour intramolecular cross-linking between adjacent thiols. Intriguingly, most of the key intracellular targets for this reaction have been identified to include the main REDOX regulatory systems in the mitochondria, including thioredoxin and peroxiredoxin systems and the adenine nucleotide transporter, all of whose function is adversely affected. The net result is the activation of several independent pathways including ROS production to facilitate the induction of apoptosis. One main pathway operates via the opening of the MPTP, the other via activation of ASK1 kinase, and the JNK/Bid/Bax pathway of channel formation in MOM. In the case of APL, cell selectivity for sensitive responses to these drugs is facilitated by selective transport systems such as provided by the AQP9 transporter. Low MDR levels present in dividing endothelial cells also provides selective targeting by specially substituted phenylarsenic-containing compounds like p-GSAO, leading to decreased blood supply into tumors, with some toxicity to cancer cells, but little toxicity on normal cells. Hence, a combination of selective delivery and retention provides the necessary targeting of arsenic-containing compounds to tumors and provides scope for additional modifications to be made to enhance the antineoplastic activity of arsenic-containing compounds, given their range of actions and efficiency in killing cancer cells.

## Figures and Tables

**Table 1:** Common names and fully systematic (additive) names for Arsenic oxoacid and related structures. Some organic derivative names still contain the word “acid,'' as in the following derivatives of arsonic acid = H_2_AsHO_3_ = [AsHO(OH)_2_], *for example*, PhAsO(OH)_2_ phenylarsonic acid.

Example of alternative names for Arsenic species used			
Common name	Abbreviation	IUPAC Fully systematic additive name	Chemical formula

Arsenite, Arsenous acid, Arsorous acid	As(III), As^III^	Trihydroxidoarsenic	H_3_AsO_3_ = [As(OH)_3_]
Arsinious acid	As(III), As^III^	Dihydrohydroxidoarsenic	HAsH_2_O = [AsH_2_(OH)]
Arsonous acid	As(III), As^III^	Hydridodihydroxidoarsenic	H_2_AsHO_2_ = [AsH(OH)_2_]
Arsenate, Arsenic acid, Arsoric acid	As(V), As^V ^	Trihydroxidooxidoarsenic	H_3_AsO_4_ = AsO(OH)_3_
Arsinic acid	As(V), As^V ^	Dihydridohydroxidooxidoarsenic	HAsH_2_O_2_ = [AsH_2_O(OH)]
Arsonic acid	As(V), As^V ^	Hydridodihydroxidooxidoarsenic	H_2_AsHO_3_ = [AsHO(OH)_2_]
Monomethylarsonic acid	MMA(V), MMA^V^	Methanedihydroxidooxidoarsenic	CH_3_AsO(OH)_2_
Monomethylarsonous acid	MMA(III), MMA^III^	Methanedihydroxidoarsenic	CH_3_As(OH)_2_
Dimethylarsinic acid	DMA(V), DMA^V^	Dimethanehydroxidooxidoarsenic	(CH_3_)_2_AsO(OH)
Dimethylarsinous acid	DMA(III), DMA^III^	Dimethanehydroxidoarsenic	(CH_3_)_2_AsOH
